# Relationship between skeletal muscle index at the third lumbar vertebra with infection risk and long-term prognosis in patients with acute-on-chronic liver failure

**DOI:** 10.3389/fnut.2023.1327832

**Published:** 2024-01-10

**Authors:** Juan Wang, Jinjia Bai, Huimin Wang, Guofen Xu, Ruoyu Yao, Jing Li, Wenrui Zhang, Han Wang, Jia Yao, Xiaojing Ren

**Affiliations:** ^1^Department of Gastroenterology, Third Hospital of Shanxi Medical University (Shanxi Bethune Hospital), Shanxi Medical University, Taiyuan, China; ^2^Endoscopy Center, Second Hospital of Shanxi Medical University, Shanxi Medical University, Taiyuan, China; ^3^Department of Gastroenterology, Jincheng General Hospital, Shanxi Medical University, Taiyuan, China

**Keywords:** acute-on-chronic liver failure, sarcopenia, low skeletal muscle index, infection, long-term survival rate

## Abstract

**Objective:**

Infection is a major cause of increased mortality in patients with acute-on-chronic liver failure (ACLF). This study aims to examine the potential correlation of the skeletal muscle index at the third lumbar vertebra (L3-SMI) with infections among ACLF patients and to evaluate its impact on the long-term survival.

**Methods:**

This retrospective study included 126 patients who underwent abdominal computed tomography (CT) and were diagnosed with ACLF at our center between December 2017 and December 2021. L3-SMI was calculated using CT, and the clinical and biochemical data as well as MELD scores were also collected, so as to analyze the relationship between L3-SMI and infections in ACLF patients and the impact on long-term prognosis.

**Results:**

Of the 126 ACLF patients enrolled, 50 had infections. In the multivariate logistic regression analysis, both L3-SMI [odds ratio (OR) = 0.89, 95% confidence interval (*CI*) = 0.81 – 0.97, *P* = 0.011] and hepatic encephalopathy (OR = 8.20, 95% *CI* = 1.70 – 39.59, *P* = 0.009) were independently associated with the risk of infection development. The overall survival (OS) estimates were obtained using Kaplan-Meier curves, and it was found that patients in the lowest tertile of L3-SMI had significantly lower 3-month, 6-month, 1-year, and 2-year survival rates than those in the highest tertile (*P* = 0.014; log-rank test).

**Conclusion:**

Low L3-SMI is an independent risk factor for the development of infections and significantly influences the long-term survival in ACLF patients.

## 1 Introduction

Acute-on-chronic liver failure (ACLF) is a clinical syndrome characterized by acute deterioration of liver function on the basis of chronic liver disease. It has a high case-fatality rate, with the 28- and 90-day mortality rates being up to 25% and 40%, respectively (1, 2). It was found that 52.2% of ACLF patients died of comorbid infection(s) (3). Identifying the risk factors that lead to infections in ACLF patients may aid in the formulation of multidisciplinary treatment protocols, ultimately reducing patient mortality rates.

Previous studies have demonstrated that hepatic encephalopathy, hepatorenal syndrome, and higher MELD scores increase the susceptibility to infection in patients with ACLF (4–6). However, these factors primarily rely on liver function indicators (7), and there is a lack of indicators for evaluating the association of general nutritional status with infections. Evidence suggests that nutritional status is closely associated with infections (8). A recent study shows that sarcopenia is a highly predictive nutritional indicator for the occurrence of hospital-acquired infections (9). Sarcopenia is a syndrome characterized by low skeletal muscle mass and progressive decline in strength and function (performance) with age (10, 11). Sarcopenia significantly increases the risk of developing infections in various conditions, including type 2 diabetes, post-gastric cancer surgery, and post-heart transplantation (12–14). Furthermore, critically ill cirrhotic patients with sarcopenia have a higher incidence of sepsis (15). Kaido et al. (16) found that low skeletal muscle index (SMI) was an independent risk factor for bacteremia after living donor liver transplantation. However, no study has yet demonstrated the effects of L3-SMI on infection and long-term survival in ACLF patients.

Patients with ACLF are often found to have sarcopenia (17–19). The SMI at the third lumbar vertebra (L3-SMI) reflects the skeletal muscle mass of the body and is also an important indicator for the diagnosis of sarcopenia (20, 21). Therefore, we assumed that L3-SMI might be a risk factor for infections and affect survivals in ACLF patients. The purpose of this study was to examine the potential correlation of L3-SMI with infections among ACLF patients, as well as to evaluate its impact on the long-term survival.

## 2 Materials and methods

### 2.1 Patients

**FIGURE 1:**
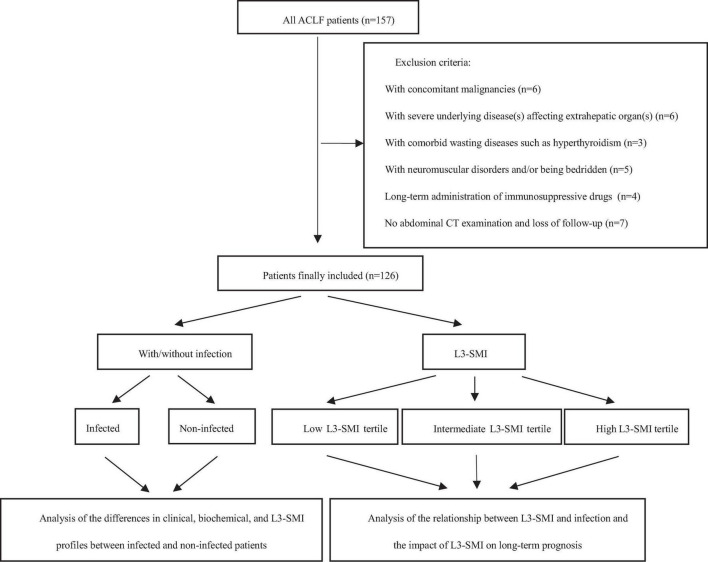
The flowchart of patient enrollment.

A retrospective cohort study was conducted on ACLF patients aged ≥ 18 years who were hospitalized at Shanxi Bethune Hospital from December 2017 to December 2021. The inclusion criteria were as follows: (a) aged ≥ 18 years; (b) underwent computed tomography (CT) within 2 weeks before and after hospitalization; and (c) diagnosed with ACLF according to the diagnostic criteria of ACLF defined by the Asian Pacific Association for the Study of the Liver (APASL) (22). Patients with one of the following conditions were excluded: (a) severe underlying disease(s) affecting extrahepatic organ(s), such as respiratory failure and/or heart failure; (b) concomitant malignancies; (c) comorbid wasting diseases such as hyperthyroidism and active tuberculosis; (d) neuromuscular disorders and/or being bedridden; and (e) long-term administration of corticosteroids and other immunosuppressant drugs. A flowchart illustrating patient inclusion is shown in Figure 1.

All patient data were obtained from the electronic medical record, and all patients were followed up every 6 months after discharge from the hospital. The scheduled follow-up duration was 4 years. Follow-up information was collected through telephone interviews. The study was conducted in accordance with the Declaration of Helsinki, and the research protocol was approved by the Shanxi Bethune Hospital. Written informed consent was obtained from all patients or their families prior to participating in the study.

### 2.2 Clinical data

The basic demographic data and clinical information of the patients, including gender, age, body height, body weight, etiology, and comorbidities (e.g., ascites and hepatic encephalopathy), were collected during hospitalization. Laboratory data were also collected from each patient at the time of diagnosis, including routine blood tests, liver function test (including alanine aminotransferase, aspartate aminotransferase, and total bilirubin), renal function test (including creatinine and urea), albumin, and coagulation-related indices. The Model for End-Stage Liver Disease (MELD) score and Child-Pugh score were calculated. The death and survival information was also collected.

Acute-on-chronic liver failure patients usually have body fluid retention such as edema and ascites. In the present study, the dry weight of the ACLF patients with body fluid retention was calculated and corrected according to the clinical severity of ascites minus a certain amount of body weight (23) (mild severity 5%, moderate 10%, severe 15%, and 5% if there was peripheral edema). The body mass index (BMI) was calculated using the following formula: BMI = dry weight (kg)/height squared (m^2^).

### 2.3 CT measurements of skeletal muscle area (SMA)

Abdominal CT examinations were performed in all patients within 2 weeks of admission. L3 intervertebral disk planar imaging was selected. Image analysis software (syngo.via Siemens AG) was used to calculate the sum of the cross-sectional areas of the skeletal muscles at the L3 level, including psoas major, erector spinae, transversus abdominis, internal abdominal oblique, external abdominal oblique, and quadratus lumborum. The SMA of the L3 cross-section was evaluated by two imaging physicians independently. When there was disagreement, a third physician was involved to reach an agreement. L3-SMI was calculated as follows: SMA at the L3 level divided by the square of height (cm^2^/m^2^) (24). In this study, patients were divided into gender-stratified L3-SMI tertiles to mitigate the known influence of gender on the outcome. Each tertile has a comparable proportion of males and females (25).

### 2.4 Diagnosis of infections

Pathogens were actively detected in the enrolled patients through the following examinations and tests: (1) history of an infection; (2) physical examination focusing on signs suggestive of an infection; (3) laboratory tests: such as erythrocyte sedimentation rate, C-reactive protein, white blood cell (WBC) count, neutrophil-to-lymphocyte ratio (NLR), and procalcitonin; (4) analyses of ascitic fluid analysis, pleural fluid, and biochemistry; and (5) Chest X-ray or chest CT. Cultures of blood, urine, stool, sputum, ascites, pleural fluid, or purulent secretions were carried out to search for pathogenic microorganisms in cases of suspected co-infection (26).

Spontaneous bacterial peritonitis (SBP) was defined by the presence of ≥ 250 polymorphonuclear cells (PMN)/mm^3^ in ascites. Pneumonia was defined as the presence of radiologic evidence of pulmonary consolidation plus at least two of the following criteria: fever above 38°C or temperature below 35°C; dyspnea; cough and sputum production; pleuritic chest pain; or signs of pulmonary consolidation on imaging. Urinary, biliary, and gastrointestinal tract infections were diagnosed by evaluating symptoms, biochemical and imaging parameters that met the established criteria (27). Positive blood culture result without a recognized site of infection was defined as spontaneous bacteremia.

According to the site of infection, standard guidelines and the results of cultures (if available), all patients with infection were assessed by infectious disease experts with expertise in nosocomial infections.

### 2.5 Statistical analysis

Statistical analysis was performed using IBM SPSS Statistics software package (version 26) (IBM Corp, Armonk, NY, USA). Demographic data, clinical features, and laboratory findings were analyzed for patients in the L3-SMI tertiles. Continuous data with normal distribution are presented as mean ± standard deviation (SD) and analyzed using one-way ANOVA; non-normal variables are reported as median [interquartile range (IQR)] and analyzed using Kruskal-Wallis test. Categorical data are presented as percentages and analyzed using Pearson’s chi-square test.

Differences in demographic characteristics, laboratory data, and L3-SMI between infected and uninfected patients were compared using unpaired *t*-tests and Mann-Whitney *U* tests (for continuous variables) or using either Pearson’s chi-square tests or Fisher’s exact tests (for categorical variables). Univariate and multivariate logistic regression analyses were conducted to investigate the risk factors associated with infections in ACLF patients. Variables that showed a *P* value of less than 0.05 in the univariate analysis were included in the multivariate analysis. Furthermore, to investigate the association between L3-SMI tertiles and infections, logistic regression was used to calculate the unadjusted odds ratio and the adjusted odds ratio of L3-SMI tertiles to infections.

Overall survival estimates were obtained using the Kaplan-Meier curves. Survival analyses were carried out to compare L3-SMI tertiles using log-rank tests. Survival rates of 3, 6, 12, and 24 months were reported.

Significant predictors of mortality in patients evaluated for ACLF were determined using univariate and multivariate Cox proportional hazard models and the results were reported as hazard ratio (HR) and 95% CI. Baseline factors known to associate with mortality of patients with ACLF including age, cirrhosis, laboratory data, MELD score, Child-Pugh score, ascites, hepatic encephalopathy as well as L3-SMI were included in univariate analysis. Variables with *P* < 0.10 in the univariate analysis were included in the multivariate model. A two-sided *P* value of < 0.05 was considered statistically significant.

## 3 Results

### 3.1 Characteristics of the study population

A total of 126 patients diagnosed with ACLF, with a mean age of 50 ± 11 years, were included in this study. Most of them were males (*n* = 80, 63%). ACLF was most commonly caused by hepatitis B (*n* = 56, 44.4%), followed by alcohol-associated hepatitis (*n* = 36, 28.6%). Compared to patients with high L3-SMI, patients with low L3-SMI exhibited lower BMI (21.45 ± 2.97 kg/m^2^ *vs.* 25.64 ± 3.49 kg/m^2^, *P* = 0.001), higher Child-Pugh score (11.48 ± 1.47 *vs.* 9.95 ± 1.96, *P* = 0.023), and higher MELD score (26.75 ± 8.96 *vs.* 21.08 ± 6.25, *P* = 0.041). Patients with low L3-SMI had a higher incidence of ascites in terms of the complications (90.5% *vs.* 52.4%, *P* = 0.019), and were significantly more likely to develop infections than those with high L3-SMI (61.9% *vs.* 19.0%, *P* = 0.018). In addition, low L3-SMI was associated with a higher mortality (61.9% *vs.* 23.8%, *P* = 0.006). The characteristics of patients in the L3-SMI tertiles are shown in Table 1.

**TABLE 1:** Characteristics of enrolled ACLF patients stratified by L3-SMI tertiles (*n* = 126).

Variables	Tertile 1 (*n* = 42)	Tertile 2 (*n* = 42)	Tertile 3 (*n* = 42)	*P*-value
Age (years)	51.62 ± 8.41	50.67 ± 12.54	48.29 ± 12.19	0.613
Weight (kg)	60.58 ± 8.10	61.54 ± 9.41	71.34 ± 10.90^b^	<0.001
BMI (kg/m^2^)	21.45 ± 2.97	22.93 ± 3.03	25.64 ± 3.49^b^	0.001
**Etiology**				
HBV (%)	16 (38.1)	18 (42.9)	22 (52.4)	0.638
Alcohol (%)	18 (42.9)	8 (19.0)	10 (23.8)	0.195
Autoimmune (%)	2 (4.8)	4 (9.5)	6 (14.3)	0.864
Others (%)	6 (9.5)	12 (33.3)	4 (9.5)	0.343
**Serum Indexes**				
RBC (10^12^/L)	3.26 ± 0.96	3.79 ± 0.74	3.32 ± 0.90	0.110
WBC (10^9^/L)	7.50 (5.10, 11.05)	5.30 (3.75, 7.30)	6.30 (4.85, 10.65)	0.130
Neutrophil (10^9^/L)	5.42 (3.29, 9.67)	3.38 (2.18, 5.16)	4.11 (2.65,7.19)	0.097
Hemoglobin (g/L)	110.10 ± 22.17	120.57 ± 20.93	113.48 ± 27.67	0.352
TBIL (mg/dL)	16.68 (11.08, 28.70)	17.11 (12.74, 22.51)	16.87 (12.39, 29.12)	0.861
Albumin (g/L)	27.07 ± 4.27	30.65 ± 6.43	28.90 ± 5.21	0.107
Scr (μmol/L)	86.40(66.70, 160.05)	77.20(68.90, 109.20)	82.05(69.50, 147.60)	0.631
INR	2.10 (1.83, 2.66)	1.82 (1.60, 2.33)	2.10 (1.66, 2.67)	0.311
Child-Pugh score	11.48 ± 1.47	9.95 ± 1.96^a^	10.57 ± 1.78	0.023
MELD scores	26.75 ± 8.96	21.08 ± 6.25^a^	25.57 ± 6.96	0.041
Ascites (%)	38 (90.5)	32 (76.2)	22 (52.4)	0.019
HE (%)	12 (28.6)	12 (28.6)	10 (23.8)	0.923
Infection (%)	26 (61.9)	16 (38.1)	8 (19.0)	0.018
Mortality (%)	26 (61.9)	8 (19.0)	10 (23.8)	0.006
L3-SMI: females (cm^2^/m^2^)	37.82 (31.33, 39.08)	42.27 (40.46, 43.08)	47.15 (46.61, 53.89)^b^	<0.001
L3-SMI: males (cm^2^/m^2^)	36.09 (34.24, 37.77)	43.83 (41.10, 45.74)	50.59 (48.18, 56.49)^b^	<0.001

*^a^P* < 0.05, Tertile 1 *vs*. Tertile 2;

*^b^P* < 0.05, Tertile 1 *vs*. Tertile 3. ALT, glutamic-pyruvic transaminase; AST, glutamic-oxalacetic transaminase; HE, hepatic encephalopathy; TBIL, total bilirubin; INR, international normalized ratio; MELD, model for end-stage liver disease; RBC, red blood cell; WBC, white blood cell; BMI, Body mass index; Scr, serum creatinine.

### 3.2 Characteristics of ACLF patients who developed infections

Of the 126 ACLF patients, 50 (40%) developed infections, among whom 16 patients were infected via more than 2 routes. Sixteen patients had more than two microbial infections. The lungs (*n* = 30) were identified as the most frequent site of infection, followed by the urinary tract (*n* = 10), bloodstream (*n* = 8), and intra-abdominal cavity (*n* = 6). There were acute cholecystitis (*n* = 6), acute cholangitis (*n* = 4), and intestinal infections (*n* = 2).

Table 2 shows the difference between ACLF patients who developed infection and those who did not. Compared with non-infected patients, infected patients had lower L3-SMI (39.40 cm/m^2^ *vs.* 45.97 cm/m^2^, *P* = 0.014) and were more likely to have comorbid hepatic encephalopathy (44.0% *vs.* 15.8%, *P* = 0.028). In addition, patients with infection had a significantly higher mortality rate than those without infection (48.0% *vs.* 26.3%, *P* = 0.012).

**TABLE 2:** Characteristics of ACLF patients with/without infection.

	With infection (*n* = 50)	Without infection (*n* = 76)	*P*-value
Age (years)	47.00 ± 10.19	52.29 ± 11.32	0.064
BMI (kg/m^2^)	22.36 ± 3.59	23.58 ± 3.68	0.221
Weight (kg)	63.30 ± 10.80	65.63 ± 10.62	0.425
WBC (10^9^/L)	7.50 (5.30, 12.20)	5.50 (4.33, 7.55)	0.012
Neutrophil (10^9^/L)	5.49 (3.55, 9.69)	3.91 (2.41, 5.28)	0.007
TBIL (mg/dL)	19.07 (10.98, 27.80)	16.67 (12.87, 24.63)	0.837
Albumin (g/L)	27.27 ± 5.11	29.93 ± 5.54	0.059
Scr (μmol/L)	100.10 (72.45, 150.75)	77.20 (66.55, 128.05)	0.139
INR	2.10 (1.66, 2.56)	1.92 (1.64, 2.69)	0.643
Child-Pugh score	11.00 (10.00, 12.00)	11.00 (9.00, 12.00)	0.230
MELD score	26.00(22.50, 29.50)	23.00 (17.00, 27.25)	0.093
HE (%)	22 (44.0)	12 (15.8)	0.028
L3-SMI (cm^2^/m^2^)	39.40 (34.68, 43.89)	45.97 (39.98, 48.28)	0.014
Mortality (%)	24 (48.0)	20 (26.3)	0.012

### 3.3 Risk factors for co-infections in ACLF patients

In the univariate and multivariate analyses, the following variables were determined to be independent risk factors for infections: hepatic encephalopathy (OR = 8.20, 95% *CI* = 1.70 – 39.59, *P* = 0.009) and L3-SMI (OR = 0.89, 95% *CI* = 0.81 – 0.97, *P* = 0.011). Table 3 summarizes the results of the binary logistic regression analysis.

**TABLE 3:** Logistic regression analysis of the risk factors of infections in ACLF patients.

	Univariate analysis		Multivariate analysis	
**Variables**	**OR (95%CI)**	***P*-value**	**OR (95%CI)**	***P*-value**
WBC (10^9^/L)	1.13 (1.00–1.27)	0.046		
Neutrophil (10^9^/L)	1.17 (1.01–1.35)	0.034		
Scr (μmol/L)	1.01 (1.00–1.02)	0.111		
Albumin (g/L)	0.91 (0.82–1.01)	0.065		
MELD score	1.03 (0.96–1.10)	0.380		
Child-Pugh score	1.27 (0.94–1.71)	0.116		
HE	4.19 (1.29–13.59)	0.017	8.20 (1.70–39.59)	0.009
L3-SMI (cm^2^/m^2^)	0.92 (0.85–0.99)	0.028	0.89 (0.81–0.97)	0.011

As shown in Table 4, low L3-SMI was associated with an increased risk of developing infections (tertile 1 vs. tertile 3: adjusted Odds ratio = 10.88, 95% CI = 1.89–62.76, *P* = 0.008).

**TABLE 4:** Unadjusted odds ratio and adjusted odds ratio for infections among ACLF patients in three L3-SMI tertiles.

	Unadjusted Odds ratio		Adjusted Odds ratio	
**L3-SMI Tertiles**	**(95%CI)**	***P* value**	**(95%CI)**	***P* value**
**First versus third**	6.91 (1.70–28.03)	0.007	10.88 (1.89–62.76)	0.008
**Second versus third**	2.62 (0.64–10.61)	0.179	6.12(0.98–38.18)	0.053

Using L3-SMI tertiles as categorical variables, multivariate analysis adjusted for white blood cells, neutrophil, creatinine, albumin, ascites, hepatic encephalopathy, MELD score, Child-Pugh score, and L3-SMI Tertiles.

### 3.4 Survival rates

**FIGURE 2:**
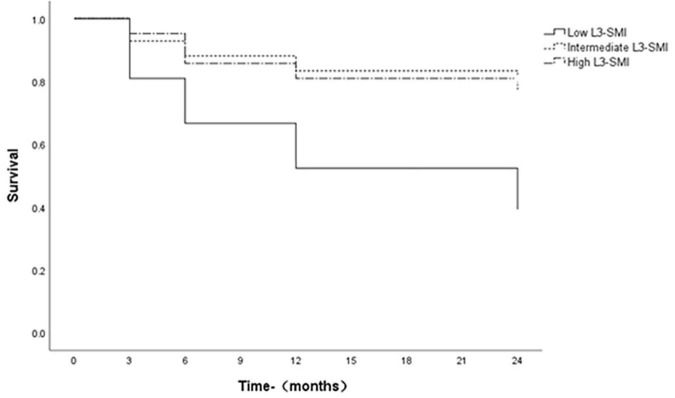
Survival curves of ACLF patients with L3-SMI tertiles. Survivals with time were assessed using Kaplan-Meier curves, which were compared using the log-rank test.

Survivals were assessed using Kaplan-Meier curves, which were compared using the log-rank test. The 3-month, 6-month, 1-year, and 2-year survival rates were 81, 67, 52, and 38%, respectively, for patients with low L3-SMI (Tertile 1), however, 93, 88, 83, and 81%, respectively, for patients with intermediate L3-SMI (Tertile 2) and 95, 86, 81, and 76%, respectively, for patients with high L3-SMI (Tertile 3), the survival rate of ACLF patients with low L3-SMI was significantly lower than that of ACLF patients with intermediate and high L3-SMI (*P* = 0.005, *P* = 0.014, log-rank test). The difference in survival rates between intermediate and high L3-SMI was not statistically significant (*P* = 0.706; Figure 2).

### 3.5 L3-SMI and long-term mortality in ACLF patients

To explore the independent relationship between L3-SMI and long-term mortality of ACLF patients, we performed univariate and multivariate Cox regression analyses of patients on the selected patients (Table 5). Univariate Cox regression analysis showed that age, creatinine, low albumin, INR, MELD score and Child-Pugh score were risk factors for long-term mortality of ACLF patients (24 months), while L3-SMI acted as a protective factor (hazard ratio (HR) = 0.876, 95%CI = 0.814–0.943, *P* < 0.001). After the variables of *P* < 0.10 in univariate analysis were included in the multivariate Cox regression model, it demonstrated that age (HR = 1.053, 95% CI = 1.004–1.104, *P* = 0.035), Child-Pugh score (HR = 1.785, 95%CI = 1.203–2.648, *P* = 0.004), L3-SMI (HR = 0.906, 95%CI = 0.841–0.976, *P* = 0.010) had an independent relationship with long-term mortality of ACLF patients.

**TABLE 5:** Univariate and multivariate Cox regression models for ACLF patients.

	Univariate analysis		Multivariate analysis	
**Variables**	**HR (95%CI)**	***P*-value**	**HR(95%CI)**	***P*-value**
Age (years)	1.043(1.005–1.082)	0.025	1.053(1.004–1.104)	0.035
Cirrhosis	1.498(0.586–3.828)	0.399		
BMI (kg/m^2^)	0.969(0.843–1.114)	0.660		
WBC (10^9^/L)	1.041(0.979–1.108)	0.200		
Hemoglobin (g/L)	0.986(0.970–1.003)	0.115		
TBIL (mg/dL)	1.002(1.000–1.004)	0.065		
INR	1.966(1.039–3.720)	0.038		
Scr (μmol/L)	1.009(1.005–1.013)	<0.001		
Albumin (g/L)	0.902(0.824–0.988)	0.027		
MELD scores	1.117(1.057–1.181)	<0.001		
Child-Pugh score	1.811(1.330–2.465)	<0.001	1.785(1.203–2.648)	0.004
Ascites	4.249(0.992–18.195)	0.051		
HE	2.118(0.904–4.965)	0.084		
L3-SMI (cm^2^/m^2^)	0.876(0.814–0.943)	<0.001	0.906(0.841–0.976)	0.010

## 4 Discussion

Acute-on-chronic liver failure is an independent clinical syndrome with a high mortality (1). Up to 80% of ACLF patients will develop bacterial infections, which are associated with worsening liver function and increased mortality (3). Therefore, it is crucial to identify risk factors for infection development and intervene promptly to minimize mortality risk. In the present study, L3-SMI was found to be an independent risk factor for infections in ACLF patients, and patients with low L3-SMI were more likely to develop infections than those with high L3-SMI. In addition, ACLF patients in the low L3-SMI quartile had significantly lower long-term survival rates than those in the high L3-SMI quartile.

The most important finding in this study is that low L3-SMI is strongly associated with the risk of developing infections in ACLF patients. Evidence suggests that sarcopenia is a risk factor for infections after liver transplantation (28). Similar to previous studies, our results indicated that the risk of developing infection was several times higher in the low L3-SMI tertile than in the high L3-SMI tertile. Further multivariate analysis showed that low L3-SMI was an independent risk factor for developing infections in ACLF patients. As for the cause of risk of infections increased by sarcopenia, we hypothesize that sarcopenia may impact the immune system, resulting in a higher risk of infection. Impaired immune response has been found in sarcopenia patients who had undergone surgery for esophageal cancer (29). Similarly, a worse systemic or local immune status in patients with sarcopenia was confirmed in patients with extrahepatic cholangiocarcinoma (30). Meanwhile, the risk of infection significantly increased in patients with sarcopenia (12, 31, 32). Similar to these studies, our study revealed that low L3-SMI (sarcopenia) affected the risk of infections in ACLF patients, The possible mechanism is that muscle cytokines such as interleukin (IL)-6 and IL-15 have been shown to be to modulate the immune system (33). However, insufficient myokine signaling in patients with sarcopenia might result in the destruction of immune system function (34). In addition, sarcopenia often represents malnutrition, several studies have proved that malnutrition lead to decrease of immune response (35, 36). The finding that improves our understanding of the risks and outcomes of sarcopenia affecting individual patients and facilitate the development of more effective management measures.

We also demonstrated that ACLF patients with a low L3-SMI had a lower long-term survival rate. Similarly, Kaido et al. (37) used bioelectrical impedance analysis to assess sarcopenia in adult patients undergoing living liver transplantation and confirmed that the OS was lower in patients with low skeletal muscle mass. A possible explanation for the impact of L3-SMI on the prognosis of ACLF patients is that skeletal muscle influences systemic energy and protein metabolism and sarcopenia may reflect protein-energy malnutrition (PEM) (38–40). Previous studies indicated that PEM was a risk factor for poor prognosis in individuals with cirrhosis (41, 42). This study found that low L3-SMI affects the long-term survival rate of ACLF patients, strengthening the management of sarcopenia may improve the prognosis of ACLF patients. It has been shown that testosterone increased muscle mass and strength (43), and testosterone therapy increased muscle mass in male cirrhotic patients with low testosterone level (44). In addition, a leucine-enriched amino acid supplementation can increase muscle strength. Resistance exercise training is an effective intervention for preventing and even reversing sarcopenia (45). Nevertheless, more treatments to improve sarcopenia in ACLF patients warrant further investigations. In the enrollment study, we also found that second tertile MELD score and Child-Pugh score were better than third tertile. But, the results of this study indicate that low L3-SMI is an independent risk factor for infection and an influential factor for long-term prognosis. These results further validates our hypothesis that skeletal muscle plays a role in predicting infection and prognosis in ACLF patients independently of established liver factors. We will further explore this phenomenon in subsequent studies.

There are limitations in our study. First, the study was a single-center and retrospective study and sample size was small. More patients from multiple regions and centers are required in future studies. Secondly, this study assessed that low L3-SMI was one of the factors affecting the risk of death and infection in ACLF patients. However, there was a lack of comprehensive assessment of the factors affecting the mortality of ACLF. Further study should be comprehensively executed to evaluate the comprehensive risk factors including L3-SMI. Furthermore, we did not analyze the direct relationship between immune status and sarcopenia due to lack of baseline data. Finally, we did not attempt to investigate differences in microbiology or site of infection among different L3-SMI tertiles, nor did we explore the potential efficacy of antimicrobial therapy. Nevertheless, standard anti-infective therapy was administered to all infected patients.

## 5 Conclusion

In conclusion, the low L3-SMI is not only a risk factor for infections but also correlates significantly with poorer survival outcomes in ACLF patients. Therefore, the management of sarcopenia should be strengthened while treating the primary affection, which may improve the infection status and poor prognosis of ACLF. Further in-depth research is needed in the future.

## Data availability statement

The original contributions presented in this study are included in this article/supplementary material, further inquiries can be directed to the corresponding authors.

## Ethics statement

The studies involving humans were approved by the Shanxi Bethune Hospital, Third Hospital of Shanxi Medical University. The studies were conducted in accordance with the local legislation and institutional requirements. The participants provided their written informed consent to participate in this study.

## Author contributions

JW: Formal analysis, Investigation, Software, Writing – original draft, Writing – review and editing. JB: Writing – original draft, Writing – review and editing. HW: Data curation, Formal analysis, Investigation, Writing – review and editing. GX: Data curation, Formal analysis, Investigation, Methodology, Writing – review and editing. RY: Data curation, Formal analysis, Investigation, Writing – review and editing. JL: Data curation, Funding acquisition, Writing – review and editing. WZ: Data curation, Formal analysis, Investigation, Software, Writing – review and editing. HW: Data curation, Formal analysis, Investigation, Methodology, Software, Writing – review and editing. JY: Conceptualization, Data curation, Formal analysis, Funding acquisition, Investigation, Methodology, Software, Supervision, Writing – review and editing, Writing – original draft. XR: Data curation, Formal analysis, Funding acquisition, Investigation, Software, Supervision, Writing – original draft, Writing – review and editing.
